# A robust immune-related lncRNA signature for the prognosis of human colorectal cancer

**DOI:** 10.1042/BSR20220078

**Published:** 2022-07-20

**Authors:** Gongmin Zhu, Lijiao Pei, Fan Yang, Chenliang Zhang

**Affiliations:** 1 Laboratory of Molecular Targeted Therapy in Oncology, West China Hospital, Sichuan University, Chengdu, Sichuan Province 610041, PR China; 2 Department of Abdominal Oncology, Cancer Center, West China Hospital, Sichuan University, Chengdu, Sichuan Province 610041, PR China; 3 The State Key Laboratory of Biotherapy, West China Hospital, Sichuan University, Chengdu, Sichuan Province 610041, PR China; 4 Chengdu Women’s and Children’s Central Hospital, School of Medicine, University of Electronic Science and Technology of China, Chengdu, Sichuan Province 611731, China

**Keywords:** colorectal cancer, immune, long non-coding RNA, prognosis, signature

## Abstract

Background: Colorectal cancer (CRC) is one of the most prevalent malignant cancers worldwide. Immune-related long non-coding RNAs (IRlncRNAs) are proved to be essential in the development and progression of carcinoma. The purpose of the present study was to develop and validate a prognostic IRlncRNA signature for CRC patients.

Methods: Gene expression profiles of CRC samples were downloaded from The Cancer Genome Atlas (TCGA) database. Immune-related genes were obtained from the ImmPort database and were used to identify IRlncRNA by correlation analysis. Through LASSO Cox regression analyses, a prognostic signature was constructed. Functional enrichment analysis was performed by gene set enrichment analysis (GSEA). TIMER2.0 web server and tumor immune dysfunction and exclusion (TIDE) algorithm were employed to analyze the association between our model and tumor-infiltrating immune cells and immunotherapy response. The expression levels of IRlncRNAs in cell lines were detected by quantitative real-time PCR (qPCR).

Results: A 9-IRlncRNA signature was developed by a LASSO Cox proportional regression model. Based on the signature, CRC patients were divided into high- and low-risk groups with different prognoses. GSEA results indicated that patients in high-risk group were associated with cancer-related pathways. In addition, patients in low-risk group were found to have more infiltration of anti-tumor immune cells and might show a favorable response to immunotherapy. Finally, the result of qPCR revealed that most IRlncRNAs were differently expressed between normal and tumor cell lines.

Conclusion: The constructed 9-IRlncRNA signature has potential to predict the prognosis of CRC patients and may be helpful to guide personalized immunotherapy.

## Introduction

Colorectal cancer (CRC) is the third most common malignancy worldwide, with almost 1.8 million new cases and approximately 8.6 million deaths in 2018 [1]. Although the diagnosis and treatment of CRC have improved significantly in the past decade, the prognosis is still poor for patients with newly diagnosed CRC presented with distant metastasis, especially liver metastasis [2]. Meanwhile, as a heterogeneous disease, the diversity of phenotypes and prognosis of CRC present a huge challenge in making individualized clinical decisions to improve the survival rate of patients [3]. Therefore, it is urgent to establish an effective risk assessment model to identify patient subgroups with different prognoses, which may also help to find potential therapeutic targets.

In recent years, genomic approaches have been applied to investigate the underlying mechanism of cancer development and explore molecular biomarkers for cancer diagnosis [4,5]. Besides the well-recognized protein-coding genes in the human genome are proved to be involved in tumorigenesis, accumulating evidence has demonstrated that long non-coding RNAs (lncRNAs), a class of non-coding RNA with more than 200 nucleotides in length, are also closely associated with the pathogenesis of tumor including cell proliferation, apoptosis, migration, and epithelial-to-mesenchymal transition [6,7]. Therefore, these findings indicate that a large number of lncRNAs can serve as potential targets and biomarkers for the diagnosis and prognosis of malignant tumors including lung, breast, liver, and colorectal cancer [8–11].

Proverbially, the immune system plays a critical role in the development of tumors. With the advent of immunologic agents, many cancers have shown positive responses to immunotherapy [12–14]. The previous study has indicated that numerous lncRNAs show an overwhelming effect on diverse stages of cancer immunity, such as antigen release and presentation, immune cell differentiation, migration, and infiltration [15]. Lnc-SNHG1 was proved to enhance regulatory T cells (Tregs) differentiation via regulating miR-448/IDO axis, which induced Tregs mediated immunosuppression and promoted immune escape in breast cancer [16]. Likewise, LncRNA MIR17HG was reported to directly bind to PD-L1 protein for accumulation in CRC, which blocked T cells activation [17]. Therefore, the dysregulation of these immune-related lncRNAs (IRlncRNAs) may suppress immune response and promote immune escape, which contributes to the occurrence and progression of various tumors.

In the present study, based on immune-related genes from the ImmPort database, we used RNA-seq dataset from The Cancer Genome Atlas (TCGA) and two microarray datasets (GSE17536 and GSE38832) from The Gene Expression Omnibus (GEO) to develop and validate a 9-IRlncRNA signature for patients with CRC. Next, we constructed a nomogram based on the 9-IRlncRNA signature, age, and M stage to evaluate clinical significance. Finally, TIMER2.0 web server and tumor immune dysfunction and exclusion (TIDE) algorithm were employed to analyze the association between our model and tumor-infiltrating immune cells and immunotherapy response.

## Materials and methods

### Data acquisition

RNA expression data, somatic mutation data, and clinical data of colon adenocarcinoma and rectal adenocarcinoma samples were downloaded from the TCGA portal (http://portal.gdc.cancer.gov/projects). 530 samples (488 tumors and 42 normal tissues) were contained in CRC patients’ dataset. For clinical data, samples with an overall survival time of less than 30 days or any missing data were excluded, and finally, 431 samples were included for subsequent study.

The gene expression profile matrix files of GSE17536 and GSE38832 were downloaded from the GEO database (https://www.ncbi.nlm.nih.gov/geo/). Raw microarray expression data were normalized using Robust Multichip Average (RMA) and converted to Log2 pattern. Probes were annotated through the Affymetrix Human Genome U133 Plus 2.0 Array. 177 samples with overall survival (OS) and disease-specific survival (DSS) from GSE17536 and 122 samples with DSS from GSE38832 were set as external validation. Details of the TCGA cohort and the two testing cohorts were shown in Table 1.

**Table 1:** Clinical information of patients with colorectal cancer in three datasets

Character	TCGA	GSE17536	GSE38832
Age (years)			
≤65	192	83	N/A
>65	239	94	N/A
Gender			
Male	235	96	N/A
Female	196	81	N/A
Grade			
Low	N/A	150	N/A
High	N/A	27	N/A
T stage			
T1	13	N/A	N/A
T2	78	N/A	N/A
T3	296	N/A	N/A
T4	44	N/A	N/A
N stage			
N0	252	N/A	N/A
N1-2	179	N/A	N/A
M stage			
M0	365	N/A	N/A
M1	66	N/A	N/A
AJCC stage			
I-II	243	81	53
III-IV	188	96	69
Survival status			
Alive	360	104	94
Deceased	71	73	28

### Acquisition of immune-related lncRNAs

The Ensemble IDs of genes from the TCGA cohort were transformed into gene symbols via the Ensemble database (http://asia.ensembl.org/index.html). Next, mRNAs and lncRNAs were extracted from the gene matrix respectively according to their biotypes. Then, mRNAs were intersected with immune-related genes (IRGs) obtained from the ImmPort database (https://immport.niaid.nih.gov) to get IRGs in colorectal cancer samples [18]. Pearson correlation analysis was applied to identify IRlncRNAs via evaluating the correlation between the IRGs and lncRNAs expression in colorectal cancer samples (|*r*| >0.3 and *P*<0.001).

### Construction and validation of the prognostic signature based on IRlncRNAs

The TCGA cohort was used for training the model. To construct the prognostic signature, 873 IRlncRNAs were intersected with the genes from GSE17536 and GSE38832, and only the IRlncRNAs contained in all cohorts were involved in the subsequent study. The expression level of these common IRlncRNAs in three cohorts was normalized using R package “sva”. Then, the least absolute shrinkage and selection operator (LASSO) Cox proportional hazard regression with 10-fold cross-validation was applied to establish IRlncRNAs signature model for the prediction of colorectal cancer prognosis. 9-IRlncRNA signature was constructed according to the optimal λ value, and the risk score for each patient was calculated by the following algorithm: risk score = (β1 × expression of lncRNA1) + (β2 × expression of lncRNA2) + … + (βn × expression of lncRNAn). All patients were divided into a high-risk group and a low-risk group according to the cut-off value (median risk score).

To validate the 9-IRlncRNA signature, the risk score was also calculated in two testing datasets (GSE17536 and GSE38832) and the patients were classified into high- and low-risk groups based on the same cut-off value.

### Establishment and evaluation of predictive nomogram

The nomogram containing the 9-IRlncRNA signature and other independent prognostic indicators was plotted using the “rms” package of R software. The total score of each patient could be calculated via the nomogram, and then was used to predict the OS rate of 1-, 3- or 5-year. The accuracy of the nomogram was evaluated using the calibration curves and the time-dependent receiver operating characteristic (ROC) curves. Decision curve analysis (DCA) was used to compare the reliability of the nomogram with that of age, M stage, or risk group.

### Calculation of tumor mutation burden (TMB)

The somatic mutation data of CRC samples were detected using VarScan. To calculate the TMB score of each sample, all base substitutions and indels in the coding region of targeted genes were counted, and silent mutations failing to lead to an amino acid change were not counted. Then, the total number of mutations counted was divided by the exome size (approximate 38 megabases) [19].

### Bioinformatics analysis

Principal component analysis (PCA) was employed to reveal the expression pattern of samples in the TCGA cohort. Gene set enrichment analysis (GSEA) was performed using Broad Institute GSEA software 4.0.1 based on TCGA datasets containing 431 CRC patients classified into high- and low-risk groups [20]. The GO gene sets “c5.bp.v7.1.symbols.gmt”, KEGG gene sets “c2.cp.kegg.v7.1.symbols.gmt”, Reactome gene sets “c2.cp.reactome.v7.1.symbols.gmt”, and PID gene sets “c2.cp.pid.v7.1.symbols.gmt” were downloaded from Molecular Signatures Database (http://software.broadinstitute.org/gsea/msigdb/index.jsp). For each gene set analysis, permutations were performed 1000 times to acquire a normalized enrichment score (NES). A normalized *P*-value < 0.05 was considered significantly enriched. TIMER2.0 web server (http://timer.cistrome.org), which integrated six state-of-the-art algorithms, including quanTIseq, CIBERSORT, xCell, MCP-counter, TIMER, and EPIC, was used to analyze the composition of tumor-infiltrating immune cells in patients in high- and low-risk groups [21]. The TIDE algorithm was used to evaluate the predictive efficiency of the 9-IRlncRNA signature for the immunotherapy response in CRC [22].

### Cell culture

Human colorectal cancer cell lines (HCT116 and SW480) were purchased from the American Type Culture Collection (Manassas, VA, U.S.A.). Human colon epithelial cell line NCM460 was purchased from the Cell Bank of Type Culture Collection of Chinese Academy of Sciences (Shanghai, China). Dulbecco’s modified Eagle medium (DMEM) containing 10% fetal bovine serum (FBS, Gibco, U.S.A.) and 1% penicillin–streptomycin was used for the cultivation of HCT116 and SW480 cells, and Roswell Park Memorial Institute (RPMI-1640) medium containing 10% FBS and 1% penicillin–streptomycin was used for NCM460. All cell lines were allowed to grow in a 37°C incubator containing 5% CO_2_.

### RNA extraction and quantitative real-time PCR

Total RNA was extracted using Cell Total RNA Isolation kit (Foregene, Chengdu, China) following the manufacturer’s protocol, and RNA (1 μg) was reverse‐transcribed to cDNA using the PrimeScript RT reagent kit (TaKaRa, Osaka, Japan). Quantitative real-time PCR (qPCR) was conducted using the SYBR Green qPCR Supermixes (Bio-Rad) on the CFX 192 Connect Real-Time PCR system (Bio-Rad, U.S.A.). The qPCR analysis was performed in triplicate with the primers shown in Table 2. The relative expression levels were normalized to GAPDH using the 2^−ΔΔCT^ method.

**Table 2:** List of primers used in the present study

Gene	Primer sequence (5′-3′)
PCED1B-AS1	Forward: TCAAGCCAATCAGCTGACAC
	Reverse: AAACAAATGCCCTGCTTGAC
VPS9D1-AS1	Forward: ATGGGTAACCAGGGGTCAAG
	Reverse: AGTAACAGTGGTAGAGCCGAC
PCAT6	Forward: ACCCCACTTTCCAGCCTG
	Reverse: AGGGAGGCTCACGGACAC
BOLA3-AS1	Forward: ATACCCCTCGTGCTCCTGAT
	Reverse: CCGCGTGCTGGACCAT
ZNF503-AS2	Forward: AGGAAACTCACTTCAAAAGCAGC
	Reverse: AAAACCGGCACTGAGAGTCC
ZEB1-AS1	Forward: TCCCTGCTAAGCTTCCTTCAGTGT
	Reverse: GACAGTGATCACTTTCATATCC
LINC01138	Forward: TATTTACGAAAGCTGAAAGCG
	Reverse: CTGCATGGGATAGGAGAAAC
WAC-AS1	Forward: GTTCAAGGCAGAAGGCCGTG
	Reverse: GGTTCAGCGTTGTTCCCAAG
COLCA1	Forward: ACTCTGATTAGGTCGGGGGA
	Reverse: ACCCACTAGCTGCCATGTTC
GAPDH	Forward: TGGTGAAGACGCCAGTGGA
	Reverse: GCACCGTAAGGCTGAGAAC

### Statistical analysis

Statistical analyses were conducted with R software (version 4.0.1) and GraphPad Prism 5.0 software (San Diego, CA, U.S.A.). Kaplan–Meier curve was employed to reflect the survival difference between high- and low-risk groups, which was assessed by log-rank test. Univariate and multivariate Cox proportional hazard regression models were used to analyze the prognostic significance of 9-IRlncRNA signature. The performance of the 9-IRlncRNA prognostic model was evaluated by area under curve (AUC) value of ROC curve and Harrell's concordance index (C-index). Two tailed Student’s *t* test and paired *t* test were utilized to compare the statistical relevance between two groups. Quantitative data are shown as the mean ± standard deviation (SD). A two-tailed *P*-value < 0.05 was regarded as statistically significant.

## Results

### Construction of the prognostic IRlncRNAs signature

**Figure 1:**
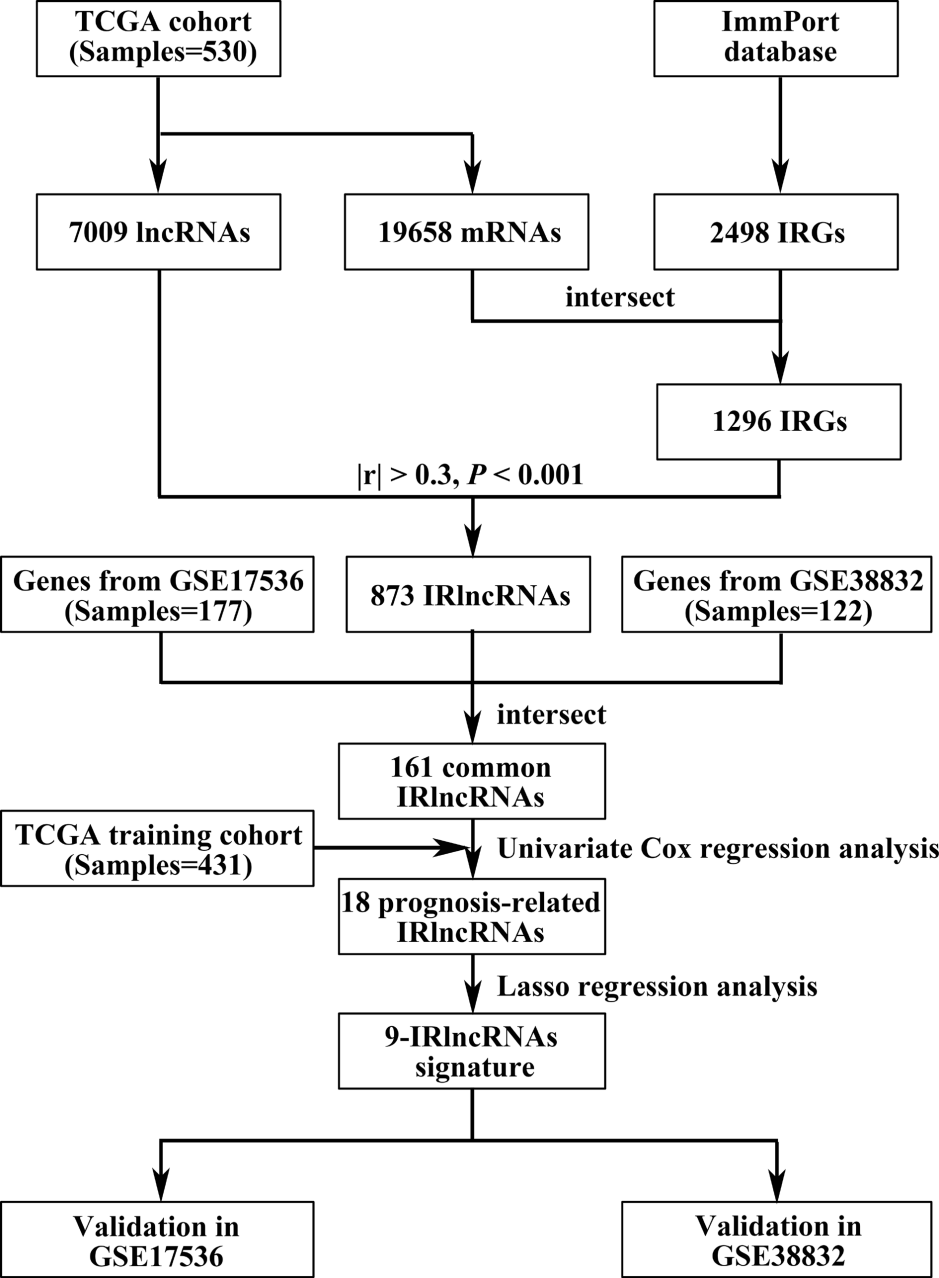
Flowchart detailing the development and validation of prognosis-related IRlncRNAs signature

**Figure 2:**
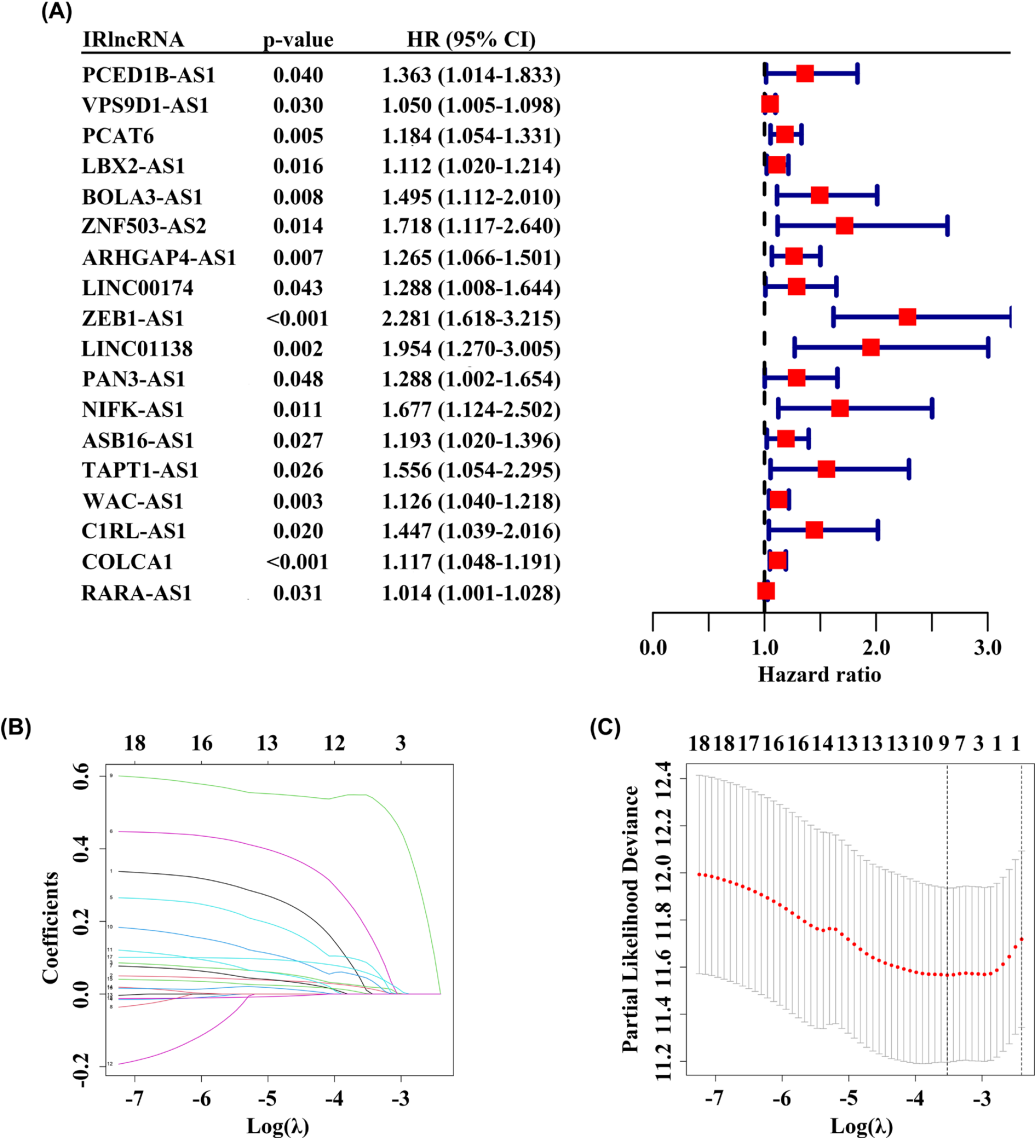
Establishment of prognostic IRlncRNAs signature (**A**) Univariate Cox regression analysis identified 18 IRlncRNAs associated with OS. (**B, C**) A 9-IRlncRNA prognostic model was constructed by a LASSO regression analysis.

To make the procedure of our study clearer, a detailed flowchart is illustrated in Figure 1. TCGA dataset was employed to set as training cohort, and a total of 161 IRlncRNAs were common among all datasets. Then, we conducted univariate Cox regression analysis to explore the prognosis-related IRlncRNAs and then 18 IRlncRNAs were identified for subsequent analysis (Figure 2A). Next, LASSO penalized Cox regression was used to establish prognostic IRlncRNAs signature, and 9 of the 18 IRlncRNAs (PCED1B-AS1, VPS9D1-AS1, PCAT6, BOLA3-AS1, ZNF503-AS2, ZEB1-AS1, LINC01138, WAC-AS1, and COLCA1) were singled out in training dataset (Figure 2B,C). Risk score of each patient was calculated according to the expression of 9 IRlncRNAs and their coefficients: risk score = (0.0109 × expression of PCED1B-AS1) + (0.0166 × expression of VPS9D1-AS1) + (0.0204 × expression of PCAT6) + (0.0867 × expression of BOLA3-AS1) + (0.1806 × expression of ZNF503-AS2) + (0.5488 × expression of ZEB1-AS1) + (0.0448 × expression of LINC01138) + (0.0011 × expression of WAC-AS1) + (0.0587 × expression of COLCA1).

### Analysis of the 9-IRlncRNA signature in the training cohort

**Figure 3:**
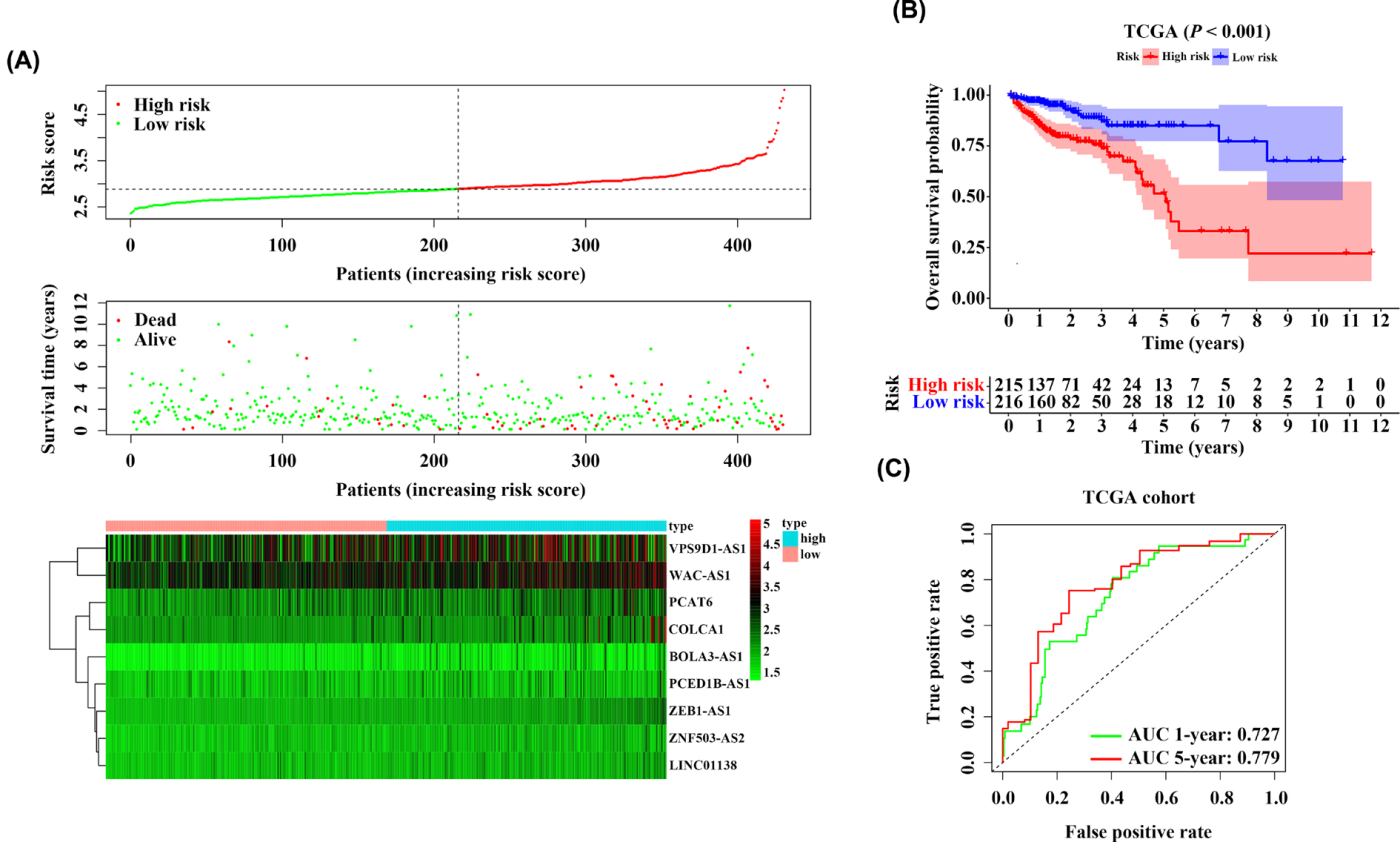
The 9-IRlncRNA signature predicts prognosis for CRC patients in TCGA training cohort (**A**) The distribution of risk score and survival status of each patient, and the heatmap of the 9 hub lncRNAs expression. (**B**) Kaplan–Meier survival curve of OS of patients with CRC in high- and low-risk group. (**C**) Time-dependent ROC curves for predicting 1- and 5-year OS.

Based on the median risk score, we classified the patients with CRC in TCGA cohort into 215 high-risk and 216 low-risk groups. As the risk score increased, both the expression of 9 IRlncRNAs and the mortality of CRC patients were elevated (Figure 3A). Similarly, Kaplan–Meier curve and log-rank test demonstrated that CRC patients with high-risk scores showed a worse OS than those with low-risk scores [hazard ratio (HR) = 3.187, 95% confidence interval (CI): 1.993–5.097, *P*<0.001] (Figure 3B). The time-dependent ROC curves showed the AUC values of 1- and 5-year OS prediction were 0.727 and 0.779, respectively, which indicated a favorable predictive value of the 9-IRlncRNA signature for the prognosis of CRC patients in the training cohort (Figure 3C).

### Validation of the 9-IRlncRNA signature in the testing cohort

**Figure 4:**
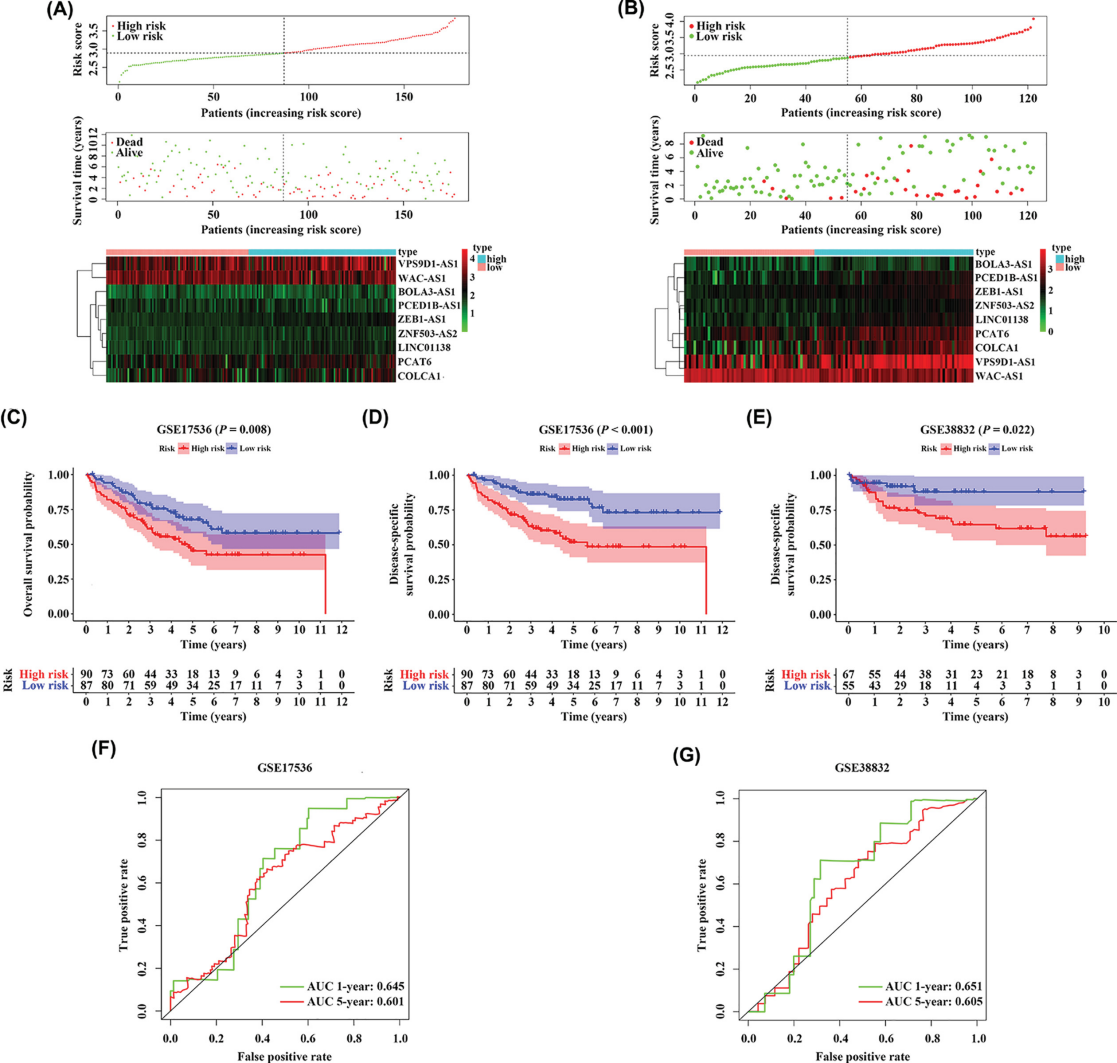
Validation of the 9-IRlncRNA signature in two testing cohorts The distribution of risk score and survival status of each patient, and the heatmap of the 9 hub lncRNAs expression in (**A**) GSE17536 cohort and (**B**) GSE38832 cohort. (**C, D**) Kaplan–Meier survival curves of OS and DSS of patients with CRC in high- and low-risk group in GSE17536 cohort. (**E**) Kaplan–Meier survival curve of DSS of patients with CRC in high- and low-risk group in GSE38832 cohort. (**F**) Time-dependent ROC curves for predicting 1- and 5-year OS in GSE17536 cohort. (**G**) Time-dependent ROC curves for predicting 1- and 5-year DSS in GSE38832 cohort.

To further validate the robustness of the 9-IRlncRNA signature in survival prediction in different cohorts, GSE17536 and GSE38832 datasets from the GEO database were set as external testing cohorts. The same cut-off value from the training cohort was used to divide the patients into high- and low-risk groups. As shown in Figure 4A,B, the higher the risk score, the more CRC patients dead. In GSE17536 dataset, we found that the OS and DSS of patients in high-risk group were obviously lower than those in low-risk group (HR = 1.865, 95% CI: 1.174–2.963, *P*=0.008; HR = 2.804, 95% CI: 1.646–4.778, *P*<0.001) (Figure 4C,D). Likewise, in GSE38832 dataset, patients with high-risk scores had a shorter DSS than those with low-risk scores (HR = 2.471, 95% CI: 1.139–5.361, *P*=0.022) (Figure 4E). These results were consistent with the finding in the training cohort. In addition, for the 9-IRlncRNA prognostic model, the AUC values of ROC curve of 1- and 5-year OS were 0.645 and 0.601 in GSE17536 dataset, and in GSE38832 dataset, the AUC values of the ROC curve of 1- and 5-year DSS were 0.651 and 0.605 (Figure 4F,G).

### The 9-IRlncRNA signature acts as an independent prognostic factor in CRC patients

**Figure 5:**
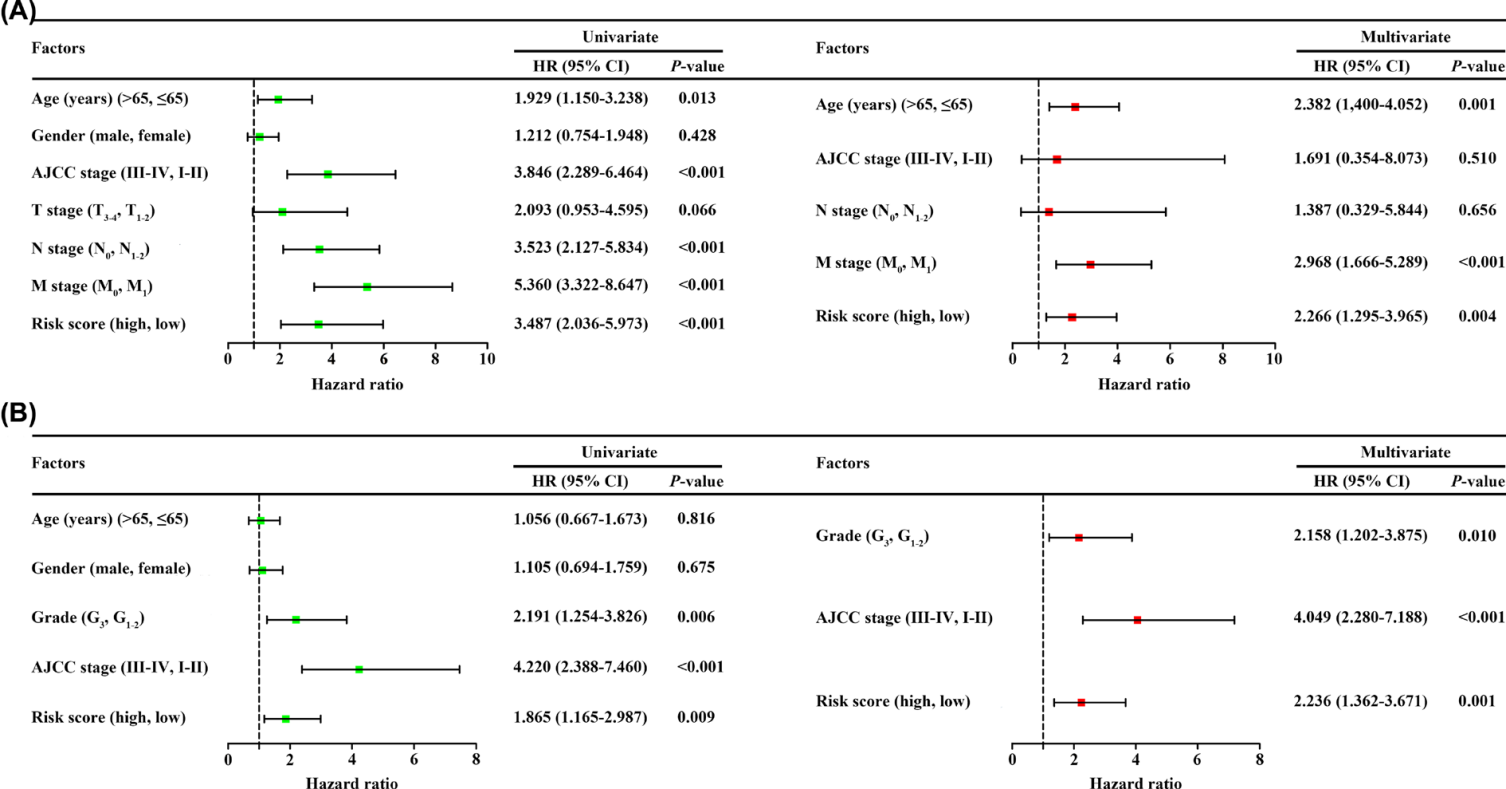
Cox regression for identifying independent prognostic factors in patients with CRC (**A**) Univariate and multivariate analyses of prognostic factors in training cohort. (**B**) Univariate and multivariate analyses of prognostic factors in testing cohort (GSE17536).

To determine that the 9-IRlncRNA signature can be an independent prognostic indicator, we employed univariate and multivariate Cox regression analyses to compare the prognostic value of the 9-IRlncRNA signature with other clinical features. The result of univariate analysis showed that age (HR = 1.929, 95% CI: 1.150–3.238, *P*=0.013), American Joint Committee on Cancer (AJCC) stage (HR = 3.846, 95% CI: 2.289–6.464, *P*<0.001), N stage (HR = 3.523, 95% CI: 2.127–5.834, *P*<0.001), M stage (HR = 5.360, 95% CI: 3.322–8.647, *P*<0.001), and 9-IRlncRNA signature risk score (HR = 3.487, 95% CI: 2.036–5.973, *P*<0.001) were significant prognostic factors. These factors were then incorporated into multivariate analysis and the results indicated that age (HR = 2.382, 95% CI: 1.400–4.052, *P*=0.001), M stage (HR = 2.968, 95% CI: 1.666–5.289, *P*<0.001), and 9-IRlncRNA signature risk score (HR = 2.266, 95% CI: 1.295–3.965, *P*=0.004) were independent prognostic factors (Figure 5A). Similarly, in testing cohort (GSE17536), 9-IRlncRNA signature risk score (HR = 2.236, 95% CI: 1.362–3.671, *P*=0.001) was also considered to be an independent prognostic indicator for OS (Figure 5B).

### Stratification analysis of the 9-IRlncRNA signature

**Figure 6:**
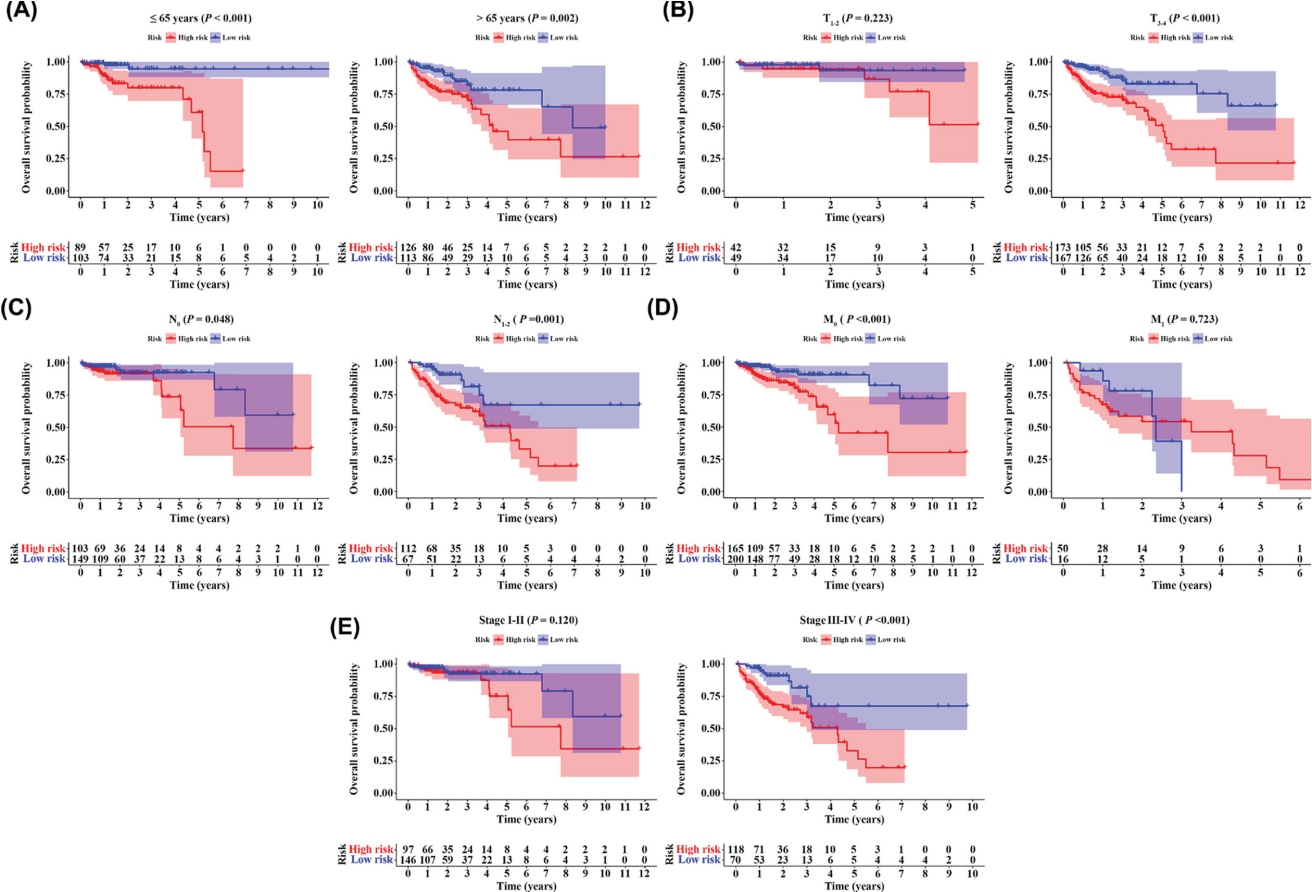
Stratification analyses Kaplan–Meier curves showed the OS of the high- and low-risk CRC patients stratified by (**A**) age, (**B**) T stage, (**C**) N stage, (**D**) M stage, and (**E**) AJCC stage.

We employed stratification analysis to confirm the prognostic value of the 9-IRlncRNA signature. Patients were stratified into different subgroups based on age (≤ 65 versus > 65 years), T stage (T_1-2_ versus T_3-4_), N stage (N_0_ versus N_1-2_), M stage (M_0_ versus M_1_), and AJCC stage (stage I-II versus stage III-IV), and were classified into high- and low-risk groups in each subgroup according to the median risk score. Interestingly, the 9-IRlncRNA signature was effective in young (HR = 5.996, 95% CI: 2.448–14.690, *P*<0.001) and elderly subgroup (HR = 2.359, 95% CI: 1.360–4.091, *P*=0.002). As for TNM stage, patients with high-risk scores predicted a poor prognosis in T_3-4_ subgroup (HR = 3.287, 95% CI: 2.003–5.393, *P*<0.001), N_0_ subgroup (HR = 2.376, 95% CI: 1.006–5.612, *P*=0.048), N_1-2_ subgroup (HR = 2.580, 95% CI: 1.458–4.566, *P*=0.001), M_0_ subgroup (HR = 3.404, 95% CI: 1.821–6.365, *P*<0.001), and stage III-IV subgroup (HR = 2.655, 95% CI: 1.517–4.645, *P*<0.001) (Figure 6A–E).

**Figure 7:**
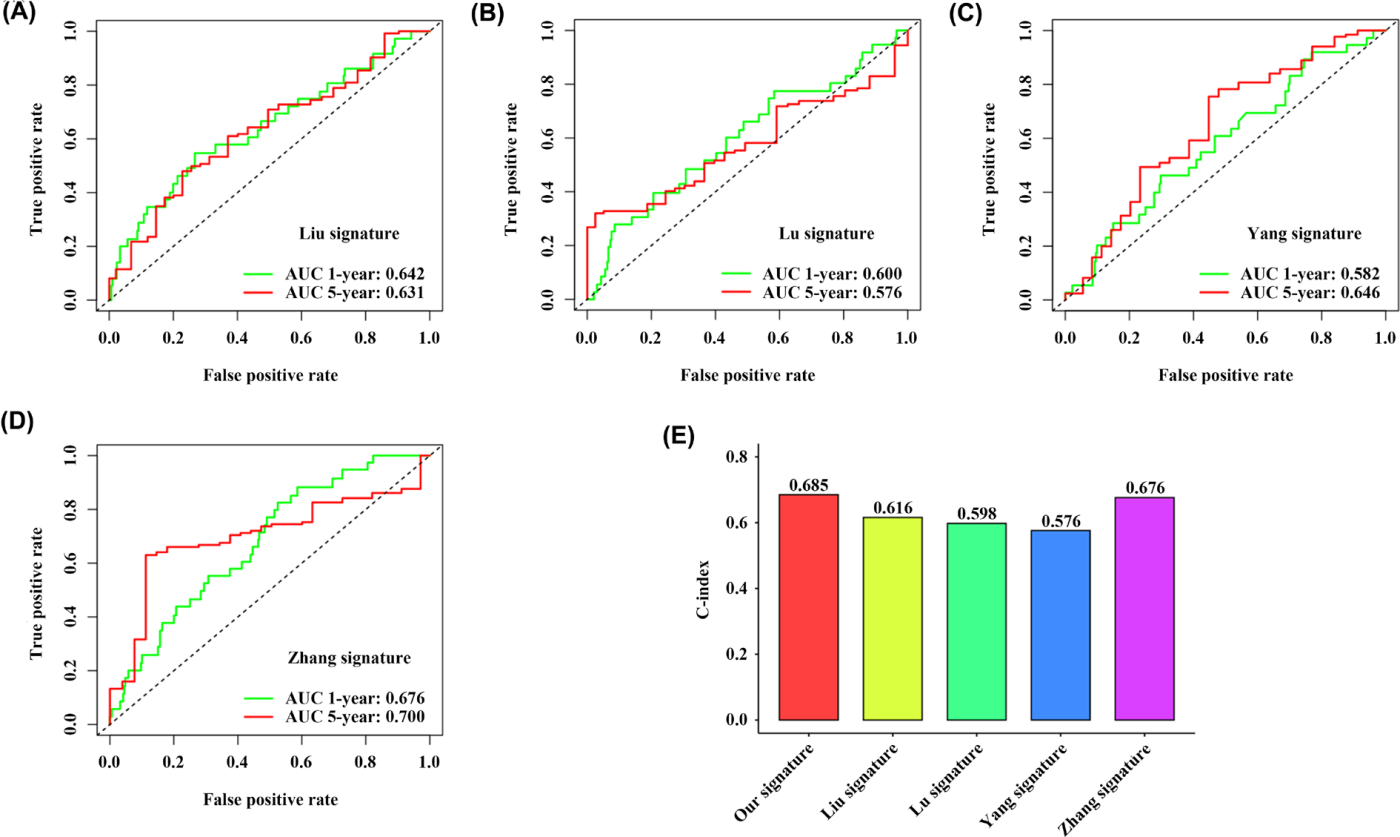
Model comparisons Comparison of 9-IRlncRNA signature with four previously proposed signatures using (**A–D**) time-dependent ROC curves for predicting 1- and 5-year OS and (**E**) C-index.

As several multi-gene signatures have been previously constructed to predict the prognosis of CRC, we evaluated their performance in parallel with our 9-IRlncRNA signature using time-dependent ROC curves and C-indexes. Obviously, the 9-IRlncRNA signature was superior to the other four models in terms of both the AUC values of 1- and 5-year OS prediction and C-index (Figures 3C and 7A–E).

### Construction of predictive nomogram

**Figure 8:**
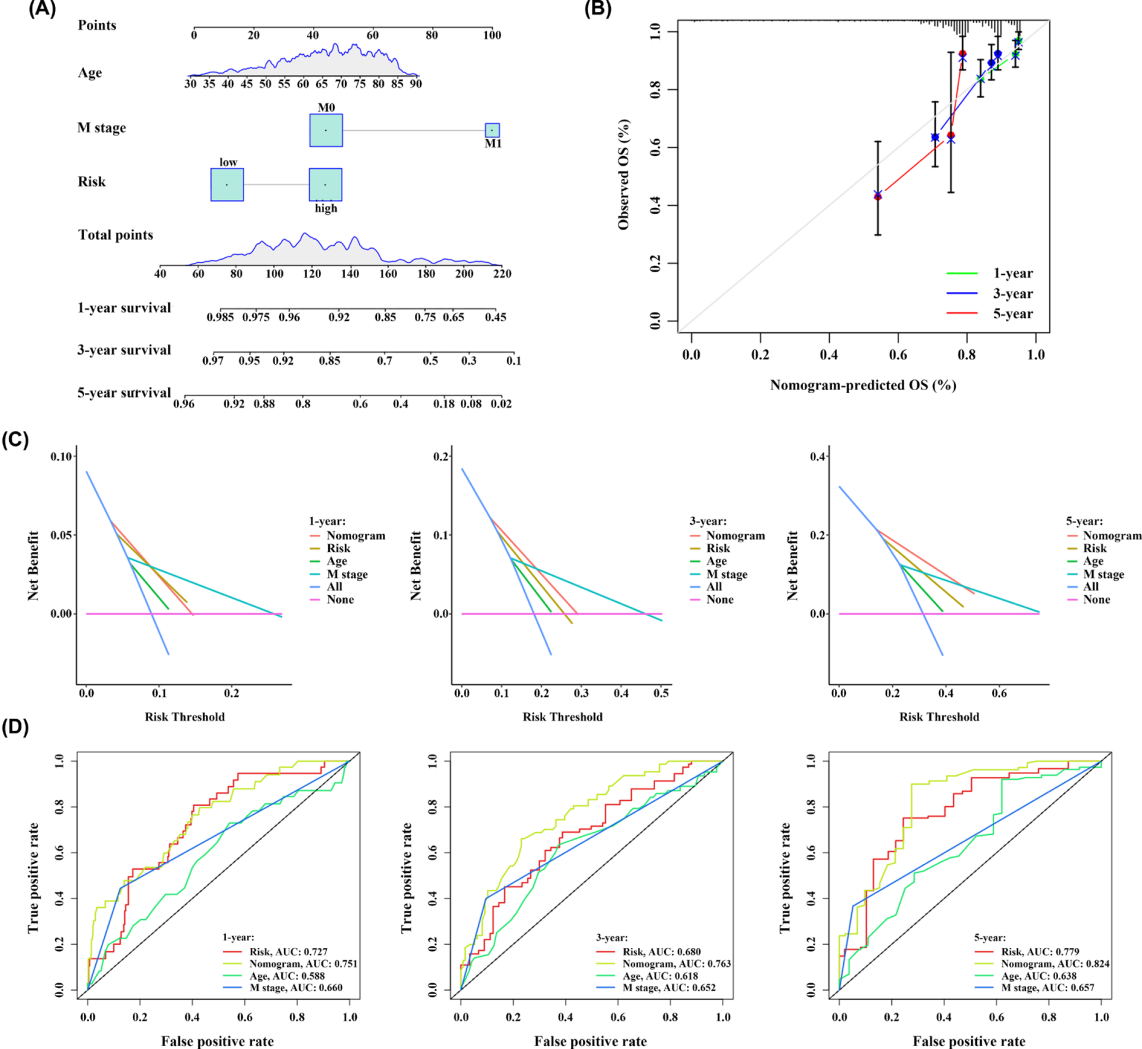
Nomogram establishment and evaluation (**A**) Nomogram for predicting survival probability at 1-, 3-, and 5-year for CRC patients. (**B**) Calibration curves for the nomogram. (**C**) DCA curves showing the comparison between the nomogram and age, M stage or risk group for predicting 1-, 3-, and 5-year OS for CRC patients. (**D**) Time-dependent ROC curves showing the comparison between the nomogram and age, M stage or risk group for predicting 1-, 3-, and 5-year OS for CRC patients.

To provide a clinical tool to predict the probability of 1-, 3-, and 5-year OS in patients with CRC, three independent prognostic factors including age, M stage and the risk score of 9-IRlncRNA signature were employed to construct a nomogram (Figure 8A). Their respective point which indicated on the top scale was added up to a total point which was corresponding to the 1-, 3-, and 5-year survival rates in the below scale. Calibration plots indicated that the nomogram predicted short-term survival (1- and 3-year) better than long-term survival (5-year) (Figure 8B). Moreover, DCA curves showed that the nomogram achieved the highest net benefit among the four factors examined (age, M stage, risk model, and nomogram) (Figure 8C). Besides, the AUC values of the nomogram at 1-, 3-, and 5-year were 0.751, 0.763, and 0.824, respectively, which also displayed the most excellent predictive performance (Figure 8D).

### Analysis of functional enrichment based on 9-IRlncRNA signature

**Figure 9:**
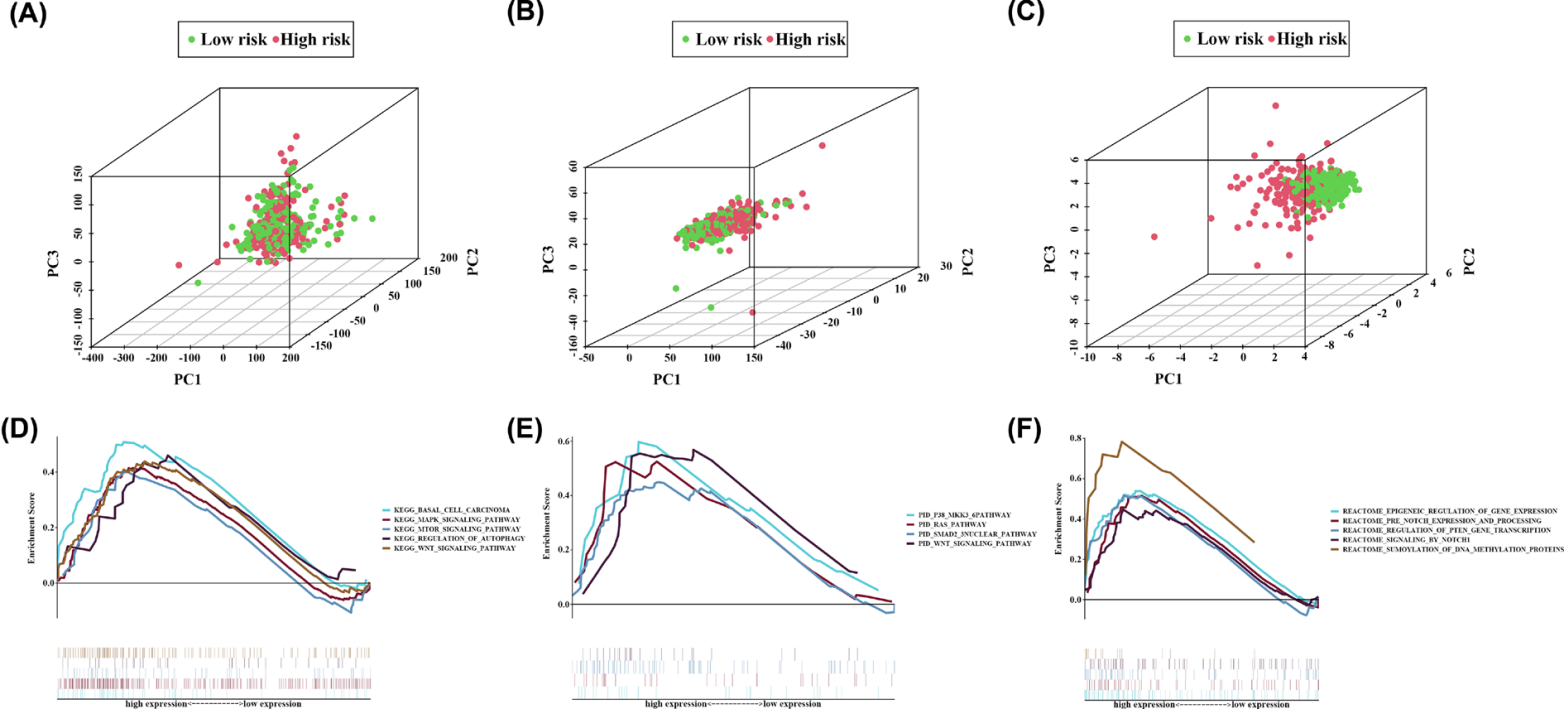
PCA and functional enrichment analysis (**A**) PCA between high- and low-risk groups based on the whole genes. (**B**) PCA between high- and low-risk groups based on the whole immune-related lncRNAs. (**C**) PCA between high- and low-risk groups based on the 9-IRlncRNA signature. GSEA based on TCGA cohort to explore the underlying mechanism of the 9-IRlncRNA signature, including gene set of (**D**) KEGG, (**E**) PID, and (**F**) REACTOME. Normalized *P*-value < 0.05.

Compared with all genes and all IRlncRNAs, the 9-IRlncRNA signature could completely distinguish high-risk patients from low-risk patients, which indicated good specificity (Figure 9A–C). To further investigate the biological process and signaling pathways involved in the 9-IRlncRNA signature, we employed GSEA to explore the pathways that were significantly altered between high- and low-risk groups. The results showed that several canonical pathways including the mTOR signaling pathway, WNT/β-catenin signaling pathway, Notch signaling pathway, and the TGF-β downstream pathway were highly enriched in the high-risk group (Figure 9D–F).

### Assessment of immune infiltration and immunotherapy-related markers with 9-IRlncRNA signature, and detection of 9 IRlncRNAs expression levels in CRC

**Figure 10:**
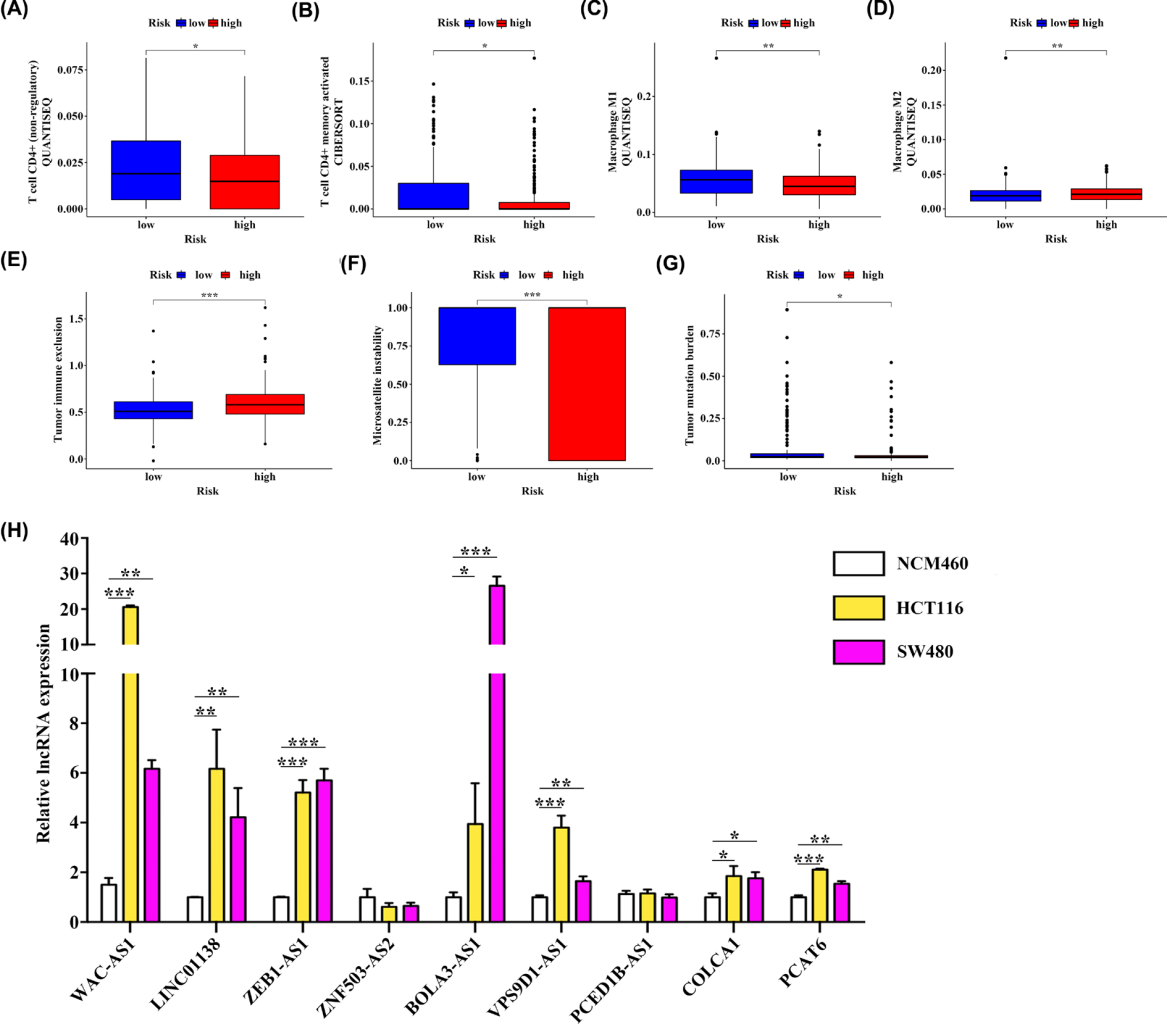
Association of the 9-IRlncRNA signature with tumor-infiltrating immune cells and its ability to predict immunotherapy response (**A–D**) The infiltration of four immune cells with significant differences in high- and low-risk groups. (**E–G**) Association of 9-IRlncRNA signature with several immunotherapy-related markers. (**H**) The qPCR analysis of expression levels of 9 IRlncRNAs in NCM460, HCT116 and SW480 cell lines; * *P*<0.05, ** *P*<0.01, and *** *P*<0.001.

We further investigated the differences in immune infiltration between high- and low-risk CRC patients. Based on quanTIseq and CIBERSORT, the results indicated that the infiltration of CD4+ T cells (non-regulatory), CD4+ memory activated T cells and M1 macrophages were higher in the low-risk group, and the content of M2 macrophages was higher in the high-risk group (Figure 10A–D). Moreover, we employed the TIDE algorithm to predict the possibility of response to immunotherapy. Interestingly, we found that low-risk group had a lower tumor immune exclusion score than high-risk group, and the microsatellite instability (MSI) score of the low-risk group was significantly higher than that of the high-risk group (Figure 10E,F). Besides, patients in low-risk group also had a higher TMB (Figure 10G).

Subsequently, we detected the expression levels of these 9 IRlncRNAs in NCM460, HCT116, and SW480 cell lines using qPCR. The result indicated that the expression levels of WAC-AS1, LINC00138, ZEB1-AS1, BOLA3-AS1, VPS9D1-AS1, COLCA1, and PCAT6 were significantly up-regulated in CRC cell lines (HCT116 and SW480), whereas the expression level of ZNF503-AS2 was lowly expressed in CRC cell lines (Figure 10H).

## Discussion

Recently, lncRNAs have been proved to play critical roles in the development and progression of many cancers [23]. In CRC, emerging evidence has indicated that lncRNAs act mostly as signaling molecules in many significant CRC-related pathways, and are frequently involved in different phases of CRC from precancerous lesions to distant metastasis [24]. A previous study has found that lncRNAs can also regulate cancer immunity, and concluded that these IRlncRNAs are a new but essential part of cancer immunotherapy and prognosis [25]. In the present study, we constructed a 9-IRlncRNAs signature that can successfully divide CRC patients into high- and low-risk groups. Meanwhile, through multivariate Cox regression analysis, the 9-IRlncRNA signature was proved to be an independent OS prognostic factor. In subsequent subgroup analysis, this prognostic signature also showed favorable stability in different subgroups. Besides, we noted that patients in high-risk group were involved in cancer-related signaling pathways such as MAPK, WNT/β-catenin, and Notch pathway, which might result in their short OS.

Through our analysis, all the nine IRlncRNAs (PCED1B-AS1, VPS9D1-AS1, PCAT6, BOLA3-AS1, ZNF503-AS2, ZEB1-AS1, LINC01138, WAC-AS1, and COLCA1) were associated with a dismal prognosis, which was consistent with their biological functions reported by previous studies. LncRNA PCED1B-AS1 was reported to promote the proliferation and inhibit the apoptosis of glioma cells via miR-194-5p/PCED1B axis [26]. However, in the present study, we found that there was no significant difference in the expression level of PCED1B-AS1 between CRC cell lines and normal colon epithelial cell lines. In non-small cell lung cancer (NSCLC), the expression of VPS9D1-AS1 was higher in NSCLC tissues than in paired adjacent tissues, and the high expression of VPS9D1-AS1 suggested an adverse prognosis [27]. Similarly, PCAT6 can bind with EZH2 which can bind to the promoter region of LATS2 and inhibit LATS2 expression in NSCLC. LATS2 overexpression can suppress cell proliferation and promote apoptosis. Therefore, lncRNA PCAT6 exerts an oncogenic function on NSCLC [28]. LncRNA ZEB1-AS1 is a well-recognized tumor-related lncRNAs and is overexpressed in several malignancies. In CRC, ZEB1-AS1 overexpression is significantly related to tumor invasion and distant metastasis, which indicates a poor OS and low recurrence-free survival rate [29]. Moreover, LINC01138 has been reported as a tumor promoter that can exert its biological functions via the tumor-related IGF2BP1/IGF2BP3-LINC01138-PRMT5 axis in hepatocellular carcinoma (HCC), which can serve as a robust biomarker and therapeutic target for HCC [30]. Recent study has reported that lncRNA WAC-AS1 is highly expressed in liver cancer tissues and cell lines, and verified that WAC-AS1 can regulate ARPP19 by sponging miR-320d to promote glycolysis and tumor proliferation [31]. Although the function of other lncRNAs remains unknown in carcinoma, their expression levels are up-regulated in CRC cell lines according to our findings, which may provide the foundation for the further exploration of the association between these IRlncRNAs and tumorigenesis. Moreover, the identification of additional targets of these nine IRlncRNAs is also a critical step to further explore their function. With the development of RNA-centric approaches, such as isolation of chromatin by RNA purification [32] and captured hybridization analysis of RNA targets [33], we will be able to identify the potential interaction targets of lncRNAs in a native context, which may deepen our understanding of lncRNA-mediated regulation of immune pathways and improve our insight in lncRNA functions.

Tumor-infiltrating immune cells are reported to be associated with tumor prognosis and have the ability to guide therapeutics [34]. In our study, patients in low-risk group had more infiltration of CD4+ T cells (non-regulatory), CD4+ memory activated T cells, and M1 macrophages. CD4+CD25+ regulatory T cells are proved to suppress antitumor response and result in tumor immune escape, while non-regulatory CD4+ helper T cells may be beneficial to the host defense against tumor [35]. CD4+ memory T cells were located in the secondary lymphoid node organs and tissues. When re-exposure to tumor antigen, CD4+ memory T cells undergo fast expansion and induce more effective and faster immune response against tumor antigen and may prevent tumor relapse [36,37]. Similarity, M1 macrophages were reported to lead to the promotion of inflammation and tumor suppression [38]. Therefore, the favorable prognosis of patients in low-risk group may be a result of the activation of various anti-tumor immune cells, and the accumulation of these immune cells may allow patients to benefit from immunotherapy. In addition to immune cell infiltration, we also found that patients in low-risk group had higher MSI and TMB scores. In CRC, immune checkpoint therapy received regulatory approval in 2017 to treat heavily mutated tumors that are mismatch-repair-deficient or harbor high levels of MSI [12]. Similarity, tumors with a higher TMB have a higher likelihood of immunotherapy response [39]. Therefore, these results further confirm that patients in low-risk group may show a favorable response to immunotherapy, and the 9-IRlncRNA signature we constructed may serve as a novel biomarker for the immunotherapy of CRC patients.

Previous studies have also constructed various lncRNA signatures to predict the prognosis of patients with CRC, but the 9-IRlncRNA we established shows more excellent performance via higher C-index and AUC values of OS [40–43]. Nevertheless, several limitations of this study should be addressed. First, the prognostic model is constructed using retrospective data, thus, the results should be further validated using prospective data or clinical trials. Second, due to the sample size was not large enough in the validation datasets, the accuracy of the prognostic model has decreased.

## Conclusion

In conclusion, we constructed a 9-IRlncRNA prognostic model to predict the prognosis of CRC patients based on the TCGA dataset. The prognostic value of this model was further validated in two external cohorts from the GEO database. Moreover, the signature was identified as an independent prognostic factor of CRC and was involved in several cancer-related signaling pathways. Furthermore, the signature was associated with tumor-infiltrating immune cells and immunotherapy-related markers. Hence, the 9-IRlncRNA signature can serve as a robust prognostic biomarker for CRC patients and may be helpful to guide personalized immunotherapy.

## Data Availability

The RNA expression data, somatic mutation data, and clinical data for training cohort are available at The Cancer Genome Atlas (TCGA) portal (http://portal.gdc.cancer.gov/projects); The data for validation cohort is available in the Gene Expression Omnibus (GEO, https://www.ncbi.nlm.nih.gov/geo/), including GSE17536 and GSE38832 datasets.

## Abbreviations

AJCC: American Joint Committee on Cancer
AUC: area under curve
C-index: Harrell’s concordance index
CI: confidence interval
CRC: colorectal cancer
DCA: decision curve analysis
DSS: disease-specific survival
GEO: Gene Expression Omnibus
GSEA: gene set enrichment analysis
HR: hazard ratio
IRG: immune-related gene
IRlncRNA: immune-related long noncoding RNA
LASSO: the least absolute shrinkage and selection operator
MSI: microsatellite instability
OS: overall survival
PCA: principal component analysis
qPCR: quantitative real-time PCR
ROC: receiver operating characteristic
TCGA: The Cancer Genome Atlas
TIDE: tumor immune dysfunction and exclusion
TMB: tumor mutation burden
Tregs: regulatory T cells
